# Cardiovascular comorbidities of atopic dermatitis: using National Health Insurance data in Korea

**DOI:** 10.1186/s13223-021-00590-x

**Published:** 2021-09-22

**Authors:** Hye Jung Jung, Dong Heon Lee, Mi Youn Park, Jiyoung Ahn

**Affiliations:** Department of Dermatology, National Medical Center, 245, Eulji-ro, Jung-gu, Seoul, 04564 Republic of Korea

**Keywords:** Atopic dermatitis, Comorbidities, Cardiovascular diseases, Metabolic syndrome

## Abstract

**Background:**

It is well known that atopic dermatitis (AD) is associated with other allergic diseases. Recentely, links to diseases other than allergic disease have also been actively studied. Among them, the results of studies regarding AD comorbidities, especially cardiovascular disease (CVD), have varied from country to country.

**Objective:**

To analyze whether the risk of CVD is different between AD patients and healthy controls using Korean National Health Insurance Data.

**Methods:**

We obtained data from 2005 to 2016 from the Korean National Health Insurance Research Database. Patients with one AD code and two AD-related tests codes were selected as AD patients, and age-and sex-matched controls to the AD patients were selected from among those without AD (1:5). Each group was investigated for accompanying metabolic syndrome (which contains hypertension, type 2 diabetes, and hyperlipidemia) and CVD (angina, myocardial infarction, peripheral vascular disease, and stroke) using ICD 10 codes.

**Results:**

The incidence of metabolic diseases and CVD were significantly different between the AD and control groups. Using multivariable Cox regression, differences were adjusted for sex, age, and other CVD and metabolic diseases. As a result, not only metabolic disease, but also the CVD risk of AD patients was significantly higher than that of the control group. Patients with AD had as significantly higher risk of hyperlipidemia (hazard ratio [HR] = 33.02, p < 0.001), hypertension (HR = 4.86, p < 0.001), and type 2 diabetes (HR = 2.96, p < 0.001). AD patients also had a higher risk of stroke (HR = 10.61, p < 0.001), myocardial infarction (HR = 9.43, p < 0.001), angina (HR = 5.99, p < 0.001), and peripheral vascular disease (HR = 2.46, p < 0.001). Besides hyperlipidemia, there was no difference in risk according to AD severity.

**Conclusion:**

Patients with AD have a greater risk of CVD than those without AD.

## Introduction

Atopic dermatitis (AD) is one of the most common inflammatory diseases in medicine, associated with a broad patient burden of skin lesions, pruritus, and both allergic and non-allergic comorbidities. The link between AD and other allergic diseases such as food allergies, allergic rhinitis, and asthma is well known [1]. Recently, in addition to allergic diseases, research has been conducted on the relationship between AD and other diseases such as depression, alopecia areata, inflammatory bowel disease, and cardiovascular disease (CVD) and metabolic syndrome [2].

Among them, the relationship between CVD, metabolic syndrome, and AD is interesting because it indicates that AD is an inflammatory disease beyond the skin and could threaten one’s life.

Regarding this comorbidity, there are some differing results on the pathogenesis depending on the country and study. An explanation for these varying results may be that observational research is more difficult to interpret in contrast to laboratory-based studies because there is a tendency to use one data source and study design per study. Accordingly, several studies have been conducted with a cross-sectional, cohort, or population-based cohort study design for AD comorbidity; and while cross-sectional studies provide information at just one point in time, cohort studies provide well described and longitudinal data [3]. No large-scale research about AD comorbidity has been done in Korea yet.

It is well known that psoriasis is related to CVD and metabolic syndrome and psoriasis is another common chronic inflammatory skin disease caused by immune dysregulation. It has already been confirmed that inflammatory mediators such as TNF-α, IL-23, and IL-17 active in psoriasis promote metabolic disease or share similar pathways [4]. It has also been confirmed that treatment of psoriasis reduces CVD risk. For example, it has been reported that patients treated with inflammation using TNF-α inhibitors have a 21% lower risk of myocardial infarction (MI) than patients treated with topical agents or phototherapy [5].

Although specific cytokines or pathways are different, these two diseases can be related due to the immune dysregulation that is involved in AD development [6].

## Methods

For the study, data satisfying the following conditions were extracted from the Korean National Health Insurance Research Database and used for analysis.

### AD patient group

Before the study, the authors conducted a validation study. It is essential to find the most accurate screening criteria for AD patients among patients with AD diagnostic codes registered in the National Health Insurance Research Database. As a result, patients who had one of the AD diagnostic codes and underwent two AD-related tests were determined to be the most appropriate [7] and were selected. The target period was from 2005 to 2016. The AD diagnosis codes used were as follows: AD (L209.000.02, L209.000.01, L209.000, and L2088.000), adult AD (L2088.004), atopic neurodermatitis (L2080.000), childhood AD (L2083.003), chronic AD (L209.000.03), and infantile AD (L2088.002).

### AD severity classification

AD was classified into clear, mild, moderate, and severe. Clear patients had a suitable diagnosis and test code but no prescriptions for drugs. Mild patients were prescribed topical steroids (TCSs) or calcineurin inhibitors (TCIs), or antihistamine agents. Moderate patients were defined as patients using TCS or TCI with antihistamine agents, and severe patients were defined as patients using systemic immune modulators.

### Control group

The control group was selected by matching patients in age and sex to the AD patient group among hemorrhoidectomy patients without AD diagnostic codes. The target period was the same as that of the patient group, and the ratio of the patient and control groups was 1: 5.

### CVD and metabolic syndrome

Both patient and control groups were investigated for hypertension (HT), diabetes mellitus (DM), and hyperlipidemia among metabolic syndromes, and angina, MI, peripheral vascular disease (PVD), and stroke among CVD. The washout period was set to 2008. From 2009 to 2016, it was determined that the patient had the disease only if they were prescribed medication twice or more in an outpatient setting or hospitalized more than once. The data from the patient group were used only after the onset of AD, and the data from the control group were used only after hemorrhoidectomy. We adjusted for age and sex for each outcome, and we additionally adjusted for each of the other 6 outcomes (e.g., for the analysis of HT, we additionally adjusted for DM, hyperlipidemia, angina, MI, PVD, and stroke), as these cardiovascular outcomes are correlated with each other. The ICD 10 codes used were as follows: I20 for angina; I21, I22, and I23 for MI; I70,I71, I72, and I73 for PVD; and I63 and I64 for stroke.

### Statistical analysis

A Mann–Whitney test was used for frequency analysis. We used a multivariable Cox regression model to calculate the multivariable-adjusted hazard ratios (HRs) and 95% confidence intervals (CIs) between AD and subsequent HT, DM, hyperlipidemia, angina, MI, PVD, and stroke. Statistical analyses were performed using SAS (Version 9.4, SAS Institute, Cary, NC), and the significance level was set at p < 0.05.

## Results

### Patient demographics

The total number of patients was 2,780,356 (49.6% male and 50.4% female). The largest proportion of patients were between 10 to 20 years old (38.5%). According to the severity classification, clear, mild, moderate, and severe cases accounted for 32.9% (49.8% male and 50.2% female), 24% (49.2% male and 50.8% female), 2.1% (50.0% male and 50.0% female), and 41% (49.7% male and 50.3% female), respectively (Table 1).

**Table 1:** Patient demographics

		N	%
Total	Total	27,80,356	100
	Male	13,79,336	49.6
	Female	14,01,020	50.4
	Under 10	7,20,298	25.9
	10 s	1,071,286	38.5
	20 s	4,51,595	16.2
	30 s	2,57,407	9.3
	40 s	1,32,198	4.8
	50 s	77,637	2.8
	60 s	43,781	1.6
	Over 70	26,154	0.9
Clear	Total	9,13,415	
	Male	4,54,539	49.8
	Female	4,58,876	50.2
	Under 10	3,03,988	33.3
	10 s	3,45,565	37.8
	20 s	1,16,756	12.8
	30 s	70,086	7.7
	40 s	36,491	4.0
	50 s	22,446	2.5
	60 s	11,719	1.3
	Over 70	6364	0.7
Mild	Total	6,67,895	
	Male	3,28,617	49.2
	Female	3,39,278	50.8
	Under 10	1,45,217	21.7
	10 s	2,93,275	43.9
	20 s	1,12,238	16.8
	30 s	55,581	8.3
	40 s	29,353	4.4
	50 s	16,338	2.4
	60 s	9653	1.4
	Over 70	6240	0.9
Moderate	Total	58,148	
	Male	29,049	50.0
	Female	29,099	50.0
	Under 10	13,486	23.2
	10 s	20,939	36.0
	20 s	12,702	21.8
	30 s	6758	11.6
	40 s	2547	4.4
	50 s	836	1.4
	60 s	481	0.8
	Over 70	399	0.7
Severe	Total	11,40,898	
	Male	5,67,131	49.7
	Female	5,73,767	50.3
	Under 10	2,57,607	22.6
	10 s	4,11,507	36.1
	20 s	2,09,899	18.4
	30 s	1,24,982	11.0
	40 s	63,807	5.6
	50 s	38,017	3.3
	60 s	21,928	1.9
	Over 70	13,151	1.2

### Trend analysis in the number of atopic patients by year

**Fig. 1:**
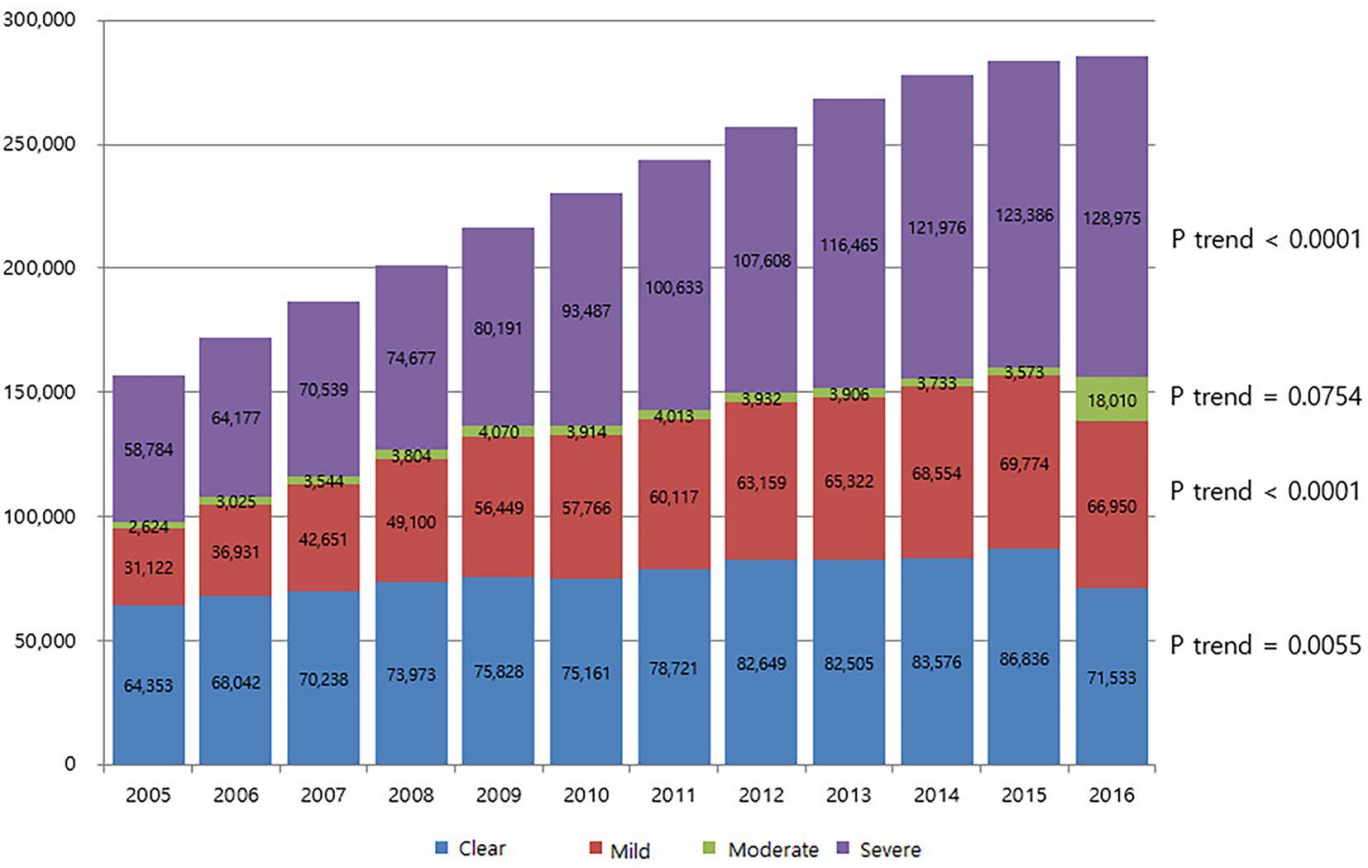
Change in the number of atopic patients by year

The total number of AD patients was 1,56,883 in 2005 and increased year by year thereafter to 2,85,468 in 2016. This increase was statistically significant. For each subgroup, those under 10 years and over 30 years increased significantly, and severe AD increased regardless of sex and age (Fig. 1).

### Frequency analysis

The proportion of AD patients among HT patients was 19.19%, and 16.81% among those without HT (p < 0.0001). The proportion of AD patients among DM, hyperlipidemia, angina, MI, PVD, and stroke was 20.02%, 23.86%, 27.16%, 24.7%, 18.94%, and 19.12%, respectively. The proportion of AD among those without DM, hyperlipidemia, angina, MI, PVD, and stroke was 16.85%, 15.94%, 16.84%, 16.85%, 16.85%, and 16.85%, respectively. Statistically significant differences were seen in DM, hyperlipidemia, angina, MI, and stroke except PVD (p = 0.1345). The results based on severity are as follows: the proportion of severe AD in HT, type 2 DM, hyperlipidemia, angina, MI, PVD, and stroke patients was 18.57%, 19.28%, 22.67%, 26.48%, 23.71%, 18.38%, 18.39%, respectively. The proportion of severe AD among those who did not have HT, type 2 DM, hyperlipidemia, angina, MI, PVD, and stroke was 14.94%, 14.99%, 14%, 14.99%, 15%, 15%, and 15%, respectively. The difference in proportions was statistically significant (Tables 2 and 3).

**Table 2:** Frequency analysis of metabolic disorders

		Without	With	p-Value
Hypertension				
AD		2,87,416 (16.81%)	3580 (19.19%)	< 0.0001
Controls		14,22,040 (83.19%)	15,072 (80.81%)	
	Controls	14,22,040 (83.19%)	15,072 (80.81%)	< 0.0001
AD	Clear	6996 (0.41%)	9 (0.05%)	
	Mild	20,262 (1.19%)	85 (0.46%)	
	Moderate	4783 (0.28%)	22 (0.12%)	
	Severe	2,55,375 (14.94%)	3464 (18.57%)	
Type 2 diabetes				
AD		2,92,987 (16.85%)	321 (20.02%)	0.0007
Controls		14,46,286 (83.15%)	1282 (79.98%)	
	Controls	14,46,286 (83.15%)	1282 (79.98%)	< 0.0001
AD	Clear	7014 (0.4%)	0 (0%)	
	Mild	20,401 (1.17%)	10 (0.62%)	
	Moderate	4824 (0.28%)	2 (0.12%)	
	Severe	2,60,748 (14.99%)	309 (19.28%)	
Hyperlipidemia				
AD		2,46,067 (15.94%)	43,746 (23.86%)	< 0.0001
Controls		12,97,450 (84.06%)	1,39,612 (76.14%)	
	Controls	12,97,450 (84.06%)	1,39,612 (76.14%)	< 0.0001
AD	Clear	6741 (0.44%)	264 (0.14%)	
	Mild	18,852 (1.22%)	1465 (0.8%)	
	Moderate	4357 (0.28%)	448 (0.24%)	
	Severe	2,16,117 (14%)	41,569 (22.67%)

**Table3:** Frequency analysis of CVD

		Without	With	p-Value
Angina				
AD		2,93,894 (16.84%)	239 (27.16%)	< 0.0001
Controls		14,50,937 (83.16%)	641 (72.84%)	
	Controls	14,50,937 (83.16%)	641 (72.84%)	< 0.0001
AD	Clear	7014 (0.4%)	0 (0%)	
	Mild	20,425 (1.17%)	4 (0.45%)	
	Moderate	4827 (0.28%)	2 (0.23%)	
	Severe	2,61,628 (14.99%)	233 (26.48%)	
Myocardial infarction				
AD		2,94,340 (16.85%)	163 (24.77%)	< 0.0001
Controls		14,52,273 (83.15%)	495 (75.23%)	
	Controls	14,52,273 (83.15%)	495 (75.23%)	< 0.0001
AD	Clear	7015 (0.4%)	0 (0%)	
	Mild	20,427 (1.17%)	6 (0.91%)	
	Moderate	4830 (0.28%)	1 (0.15%)	
	Severe	2,62,068 (15%)	156 (23.71%)	
Peripheral vascular diseases				
AD		2,93,968 (16.85%)	136 (18.94%)	0.1345
Controls		14,50,617 (83.15%)	582 (81.06%)	
	Controls	14,50,617 (83.15%)	582 (81.06%)	0.0086
AD	Clear	7016 (0.4%)	0 (0%)	
	Mild	20,423 (1.17%)	2 (0.28%)	
	Moderate	4828 (0.28%)	2 (0.28%)	
	Severe	2,61,701 (15%)	132 (18.38%)	
Stroke				
AD		2,94,139 (16.85%)	290 (19.12%)	0.0186
Controls		14,51,198 (83.15%)	1227 (80.88%)	
	Controls	14,51,198 (83.15%)	1227 (80.88%)	0.0001
AD	Clear	7014 (0.4%)	2 (0.13%)	
	Mild	20,427 (1.17%)	7 (0.46%)	
	Moderate	4830 (0.28%)	2 (0.13%)	
	Severe	2,61,868 (15%)	279 (18.39%)	

### Multivariable Cox regression analysis

**Fig. 2:**
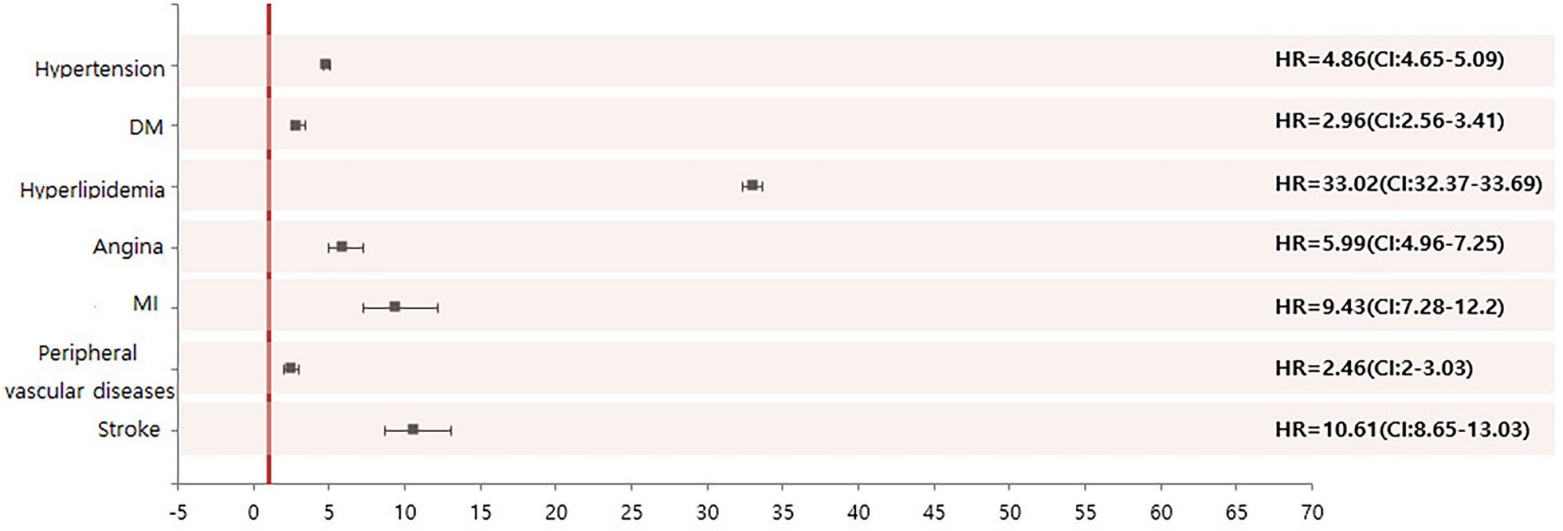
Multivariable Cox regression analysis

Using a multivariable Cox regression model, differences were adjusted for sex, age, and other CVD and metabolic diseases. As a result, the metabolic disease and CVD risk of AD patients was significantly higher than that of the control group. Patients with AD had a significantly higher risk of hyperlipidemia (HR = 33.02, p < 0.001), HT (HR = 4.86, p < 0.001), and type 2 DM (HR = 2.96, p < 0.001). AD patients also had a higher risk of stroke (HR = 10.61, p < 0.001), MI (HR = 9.43, p < 0.001), angina (HR = 5.99, p < 0.001) and PVD (HR = 2.46, p < 0.001) (Table 4 and Fig. 2).

**Table 4:** Multivariable Cox regression analysis

		HR	CI		P
HT	Total	4.86	4.65	5.09	< 0.0001
	Clear	2.61	1.36	5.02	0.004
	Mild	4.62	3.73	5.72	< 0.0001
	Moderate	4.61	3.03	7.02	< 0.0001
	Severe	4.88	4.67	5.11	< 0.0001
Type 2 DM	Total	2.96	2.56	3.41	< 0.0001
	Clear	0.00	0.00	0.00	0.937
	Mild	4.01	2.14	7.50	< 0.0001
	Moderate	3.27	0.82	13.11	0.0948
	Severe	2.94	2.54	3.39	< 0.0001
Hyperlipidemia	Total	33.02	32.37	33.69	< 0.0001
	Clear	14.93	13.22	16.85	< 0.0001
	Mild	24.05	22.80	25.38	< 0.0001
	Moderate	29.04	26.44	31.90	< 0.0001
	Severe	34.35	33.66	35.06	< 0.0001
Angina	Total	5.99	4.96	7.25	< 0.0001
	Clear	0.00	0.00	0.00	0.9434
	Mild	2.88	1.07	7.75	0.0365
	Moderate	6.49	1.61	26.18	0.0085
	Severe	6.16	5.09	7.47	< 0.0001
MI	Total	9.43	7.28	12.20	< 0.0001
	Clear	0.00	0.00	0.00	0.9408
	Mild	5.50	2.41	12.57	< .0001
	Moderate	3.92	0.55	28.15	0.1743
	Severe	10.13	7.79	13.17	< .0001
PVD	Total	2.46	2.00	3.03	< 0.0001
	Clear	0.00	0.00	0.00	0.9556
	Mild	1.44	0.36	5.79	0.6082
	Moderate	6.41	1.59	25.79	0.0089
	Severe	2.48	2.01	3.05	< .0001
Stroke	Total	10.61	8.65	13.03	< 0.0001
	Clear	18.20	4.52	73.20	< 0.0001
	Mild	10.35	4.85	22.08	< 0.0001
	Moderate	12.72	3.15	51.35	0.0004
	Severe	10.56	8.59	12.98	< 0.0001

### Multivariable Cox regression analysis according to AD severity

**Fig. 3:**
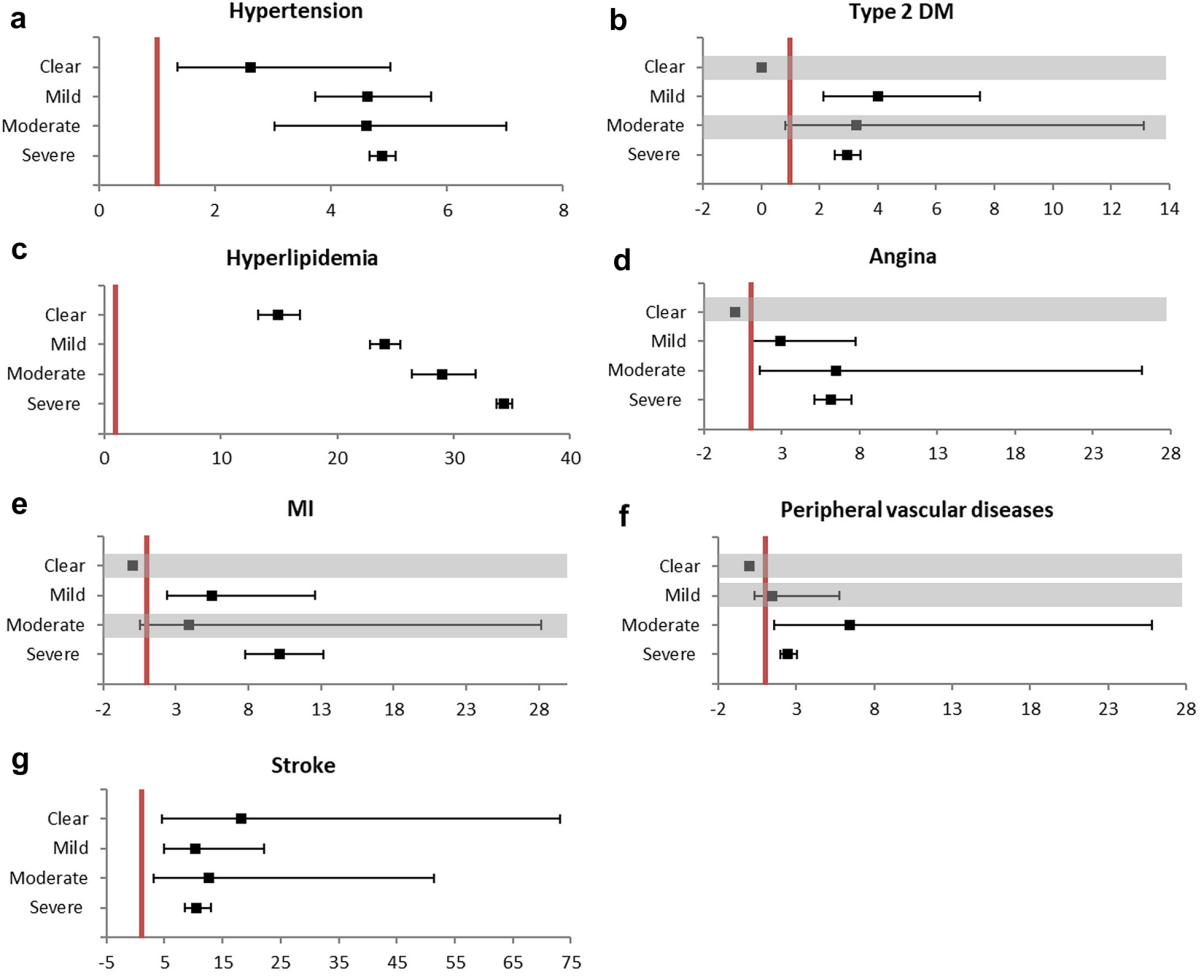
Multivariable Cox regression analysis according to the severity of AD. **a** Hypertension **b** type 2 Dm **c** hyperlipidemia **d** angina **e** MI** f** peripheral vascular diseases **g** stroke

Differences in metabolic diseases or CVD according to AD severity were analyzed. As the severity of AD increased, the risk of hyperlipidemia increased (HR = 14.93, 24.05, 29.04, and 34.35 in clear, mild, moderate, and severe cases) (p < 0.001). However, in other diseases, a consistent increase in risk according to the AD severity could not be confirmed (Table 4 and Fig. 3).

## Discussion

There are several studies on the relationship between AD and CVD. When the link between the two diseases was first noticed, studies mainly focused on establishing the existence of the link. Initially, results were often reported that the two diseases were not related [8–13]. In 2017, Standl et al. [9] investigated the relationship between AD and CVD in three groups of cohorts and whether they had genetic risk factors of CVD. As a result, the risk of angina, HT, and PVD was slightly increased in only one cohort, and the risks of other diseases, such as MI and stroke, were not different. The other two cohorts showed no difference in all diseases. In addition, differences in genetic the risk factors of CVD had not been identified. In a systemic review and meta-analysis conducted by Thyssen et al. [10] AD was only related to angina but not type 2 DM, HT, stroke, and MI.

In 2017, the Council of the International Eczema of Council reviewed the results of previous studies and argued that AD and cardiovascular morbidities are related [14]. In addition, in a population-based matched cohort study conducted in 2018, the risk of CVD in AD patients was increased, which was proportional to the severity of AD [15]. Subsequently, the relationship between the two diseases has been steadily reported [4, 14–25].

Recent research has been actively conducted on the mechanism of AD affecting CVD, in addition to elucidating their relationship. The results of how diseases affect each other can be roughly divided into two categories: direct or indirect.. Direct interaction appears primarily in the results of studies conducted in some of the United States and Asia [11, 16, 24, 26]. It is an opinion that immune dysregulation of AD directly acts as a factor to increase CVD risk. In this case, even if confounding factors are adjusted, the difference in risk is maintained [21, 27, 28]. Indirect interaction appears in results obtained mainly in Denmark, a proxy for Europe [10, 19, 20, 25, 29]. AD increases other risk factors of CVD such as sleep disturbance, depression, oral corticosteroid use, smoking, obesity, type 2 DM, HT, hyperlipidemia, and low physical activity. In this case, if the other factors are corrected, the difference in risk disappears [10, 29]. However, direct and indirect interactions are not always clearly distinguished. In a cross-sectional US population-based study conducted by Silverberg [17] in 2018, moderate to severe AD showed direct effects on DM and obesity. Additionally, it was confirmed that AD has indirect effects on high blood pressure and heart disease.

The results of our study confirmed that AD is associated with CVD after adjusting for other variables. Namely, when analyzing the association between AD and each disease, the difference from the control group was statistically significant even though corrections were made for other metabolic diseases or CVD, indicating that AD directly affects CVD and metabolic disease.

In particular, the positive correlation between CVD risk and the severity AD provides the strongest evidence for this relation. Recently, there have been reports of increased markers or similar pathways that commonly act in AD and CVD [28, 29]. They are likely to serve as the basis for direct associations between AD and CVD. It has been found that AD patients have increased levels of markers of atherosclerosis including fractalkine/CX3CL1, CCL8, M-CSF, and HGF, as well as increased levels of mediators of atherosclerosis such as E-selectin or PI3/elafin, CCL17, and IL-16, which are proportional to SCORAD [27]. In addition, it has been reported that AD patients over the age of 60 have increased atherosclerosis markers such as CCL4, CCL7, and SORT1, cardiovascular risk markers (GDF15, MPO, ST2), and factors related to cell adhesion or apoptosis compared to young AD patients healthy indivisuals of the same age [28].

Meanwhile, there are many reasons for the heterogeneity of results regarding AD and its comorbidities. First, there are differences in the way AD is diagnosed. For example, AD patients may be selected by looking at previous medical records or through questionnaires. However, depending on the diagnostic method, AD diagnosis may not be accurate. One study obtained data through three types of questionnaires, and then AD and obesity were diagnosed. When comparing the diagnosis by the three questionnaires, the results were inconsistent [30]. In addition, the accuracy of AD diagnosis has been controversial when using registered ICD10 codes as in this study. This is because the accuracy may vary depending on what criteria are used and who performs the diagnosis. This study is meaningful in that it confirmed the accuracy of diagnosis in advance through validation.

Second, research methods are also very diverse, such as cross-sectional, population-based cohorts, or patient cohort studies [3]. In 2019, Ascotte et al. [23] conducted a systemic review and meta-analysis. They analyzed the study subjects by dividing it into cross-sectional and longitudinal cohort studies. In addition, it was analyzed according to the severity of AD. The cross-sectional study showed heterogenous results without any relationship between the diseases. In the cohort study, AD patients had a higher risk of MI, ischemic stroke, angina, and heart failure. In this study, it was found that the risk of CVD increased with the severity of AD. Dermatoepidemiologic studies include cross-sectional studies, routinely collected data, case control studies, population-based cohorts, patient cohorts, and interventions. More reliable results can be obtained by going to interventions in cross-sectional studies. In such large-scale studies, it is not easy to conduct patient cohorts or intervention studies. Our study is a population-based cohort study which can obtained relatively good results using well described longitudinal data [3].

In addition to the above two reasons, the types of diseases to be investigated and confounding factors differ slightly from study to study. Besides depending on the severity of AD, the effect on comorbidities may vary. Most studies have not classified subjects according to severity, and even if classified, the criteria are not uniform.

The results of this study have various implications. First, the steady increase in AD was confirmed in the results of this study. Among them, the increase in AD over 30 years of age indicated that the study of systemic diseases that occur mainly in adults, such as CVD, is crucial for AD research. In addition, an increase in severe AD should also be noted. If the increase in comorbidities increases according to the severity, more active treatment is required. There have been only a few results from Taiwan in Asian studies, and the results from Koreans have been added to compare the differences among Asians.

A limitation of this study is that some factors important to CVD have not been adjusted for. Adjustments were made for age and sex, metabolic disorders, and other CVDs, but not for education level, physical activities, medication, obesity (body mass index), smoking, and alcohol intake. Another limitation is that it is impossible to accurately determine the causal relationship over time due to the study design, and there may be cases where an incident occurs, which is a problem that most studies have in common. Accordingly, a well-planned longitudinal prospective cohort study should be conducted to draw a more accurate conclusion.

## Conclusion

Atopic dermatitis (AD) is one of the most common inflammatory skin disease. Recently, in addition to allergic diseases, research has been conducted on the relationship between AD and other diseases especially cardiovascular disease (CVD) and metabolic syndrome. We analyzed whether the risk of CVD is different between AD patients and healthy controls using Korean National Health Insurance Data. As a result, not only metabolic disease, but also the CVD risk of AD patients was significantly higher than that of the control group. Patients with AD had as significantly higher risk of hyperlipidemia, hypertension, and type 2 diabetes. AD patients also had a higher risk of stroke, myocardial infarction, angina, and peripheral vascular disease. A well-planned longitudinal prospective cohort study should be conducted to draw a more accurate conclusion.

## Data Availability

Not applicable.
